# On Some of the Hemorrhages of Pregnancy

**Published:** 1901-09

**Authors:** A. E. Aust Lawrence

**Affiliations:** Professor of Midwifery and Diseases of Women, University College, Bristol, and Consulting Physician-Accoucheur to the Bristol General Hospital.


### ON SOME OF THE HEMORRHAGES OF PREGNANCY.

BY

A. E. Aust Lawrence, M.D.,

*Professor of Midwifery and Diseases of Women, University College, Bristol, and Consulting Physician-Accoucheur to the Bristol General Hospital.*

Read before the Bristol Medico-Chirurgical Society, May 8th, 1901.

---

I shall confine my remarks to cases of hemorrhage from the genital tract occurring during the first three months of pregnancy, as about this time there are many and special causes for it. I need hardly say that the first point we have to decide is that of pregnancy itself; and this is often one of extreme difficulty, the ordinary symptoms either being in some cases entirely absent, or so masked by other conditions as to be recognised with the greatest difficulty. In ordinary diseases a mistake in diagnosis often redounds to the doctor's credit. In midwifery this is never the case, and a mistake in the diagnosis of pregnancy is supposed to show great incapacity on the part of the doctor. The several important points in this diagnosis I shall put before you as we take the cases themselves. Most frequently the practical question we have to decide is the source of the bleeding. Is it from inside the uterus, or from the cervix or vagina? If we make an examination with a speculum we can see if it comes from inside the os uteri or external to it, either from the cervix uteri or the vaginal walls. I shall best illustrate my subject by shortly relating cases from my own practice and commenting on them.

We all know that as a rule menstruation ceases during pregnancy, but it is possible for a woman to be pregnant and yet have a periodical discharge of blood simulating menstruation, and for this blood to be seen to come from inside the os uteri. This occurred in the case I now refer to, where a married woman consulted me on account of an abdominal enlargement, which proved to be uterine, and owing to the supposed persistence of menstruation was not recognised as a case of pregnancy. She went to her full time and was delivered of a living child, and it was found at the time of confinement that she possessed a normal uterus.

Cases like this have been often recorded, but here the diagnosis of early pregnancy rests on other symptoms, chiefly on the capacity of the medical attendant to recognise the purely uterine symptoms of pregnancy; viz., the alteration in the position and shape of the body of the uterus, and the consistence of the cervix, &c.

It is possible even in a normal uterus for a woman to have one natural menstrual period after impregnation has taken place. This is often the source of error in calculating the date of confinement. For instance, a lady recently consulted me as to whether she was pregnant or not. Throughout her first pregnancy she had a periodical discharge of blood in all respects like normal menstruation. She had no sickness, yet she was pregnant. She had missed four monthly periods, and had no sickness until three days ago. The uterus was enlarged equal to four months, and the cervix soft and characteristic of pregnancy. Why she had a periodical uterine hemorrhage before I cannot say. She certainly had nothing wrong with her uterus. In neither of these two cases was the uterus bifid, which of course might account for the persistence of monthly bleeding. These cases show how important it is not to rely on one symptom as indicative of pregnancy. In one case I overlooked pregnancy because the woman had been under my care for some years with a fibroid uterus, chiefly affecting the right side, and forming a tumour equal to a four months' pregnancy. She had a regular monthly bleeding, and came to me on account of a slowly-growing left-sided swelling. The cervix uteri had not the characters of pregnancy, and there being a regular monthly flow of blood I overlooked the possibility of pregnancy, and passed a sound, thinking that the left swelling was another fibroid outgrowth. She aborted in the night, the left-sided swelling disappearing. I ought to have been suspicious of pregnancy, as the recent swelling was of a softer character than the right-sided one; and I ought to have listened over the uterus, as then I should have heard the fœtal heart, the woman being five months pregnant. Of course hearing a bruit would not have helped me, as you get this in pregnancy and in enlargements of the uterus from other causes. I offer no excuse, even that of youth, for this mistake. I mention the case as a warning to others.

There is one frequent cause of sometimes regular monthly bleeding and sometimes irregular; viz., a granular condition of the os and cervix uteri. This can be seen through a speculum, and touching with forceps makes it bleed easily. The treatment is to scarify the granular surface freely, and apply strong nitric acid. This always cures, and I have never seen an abortion produced by it. There is one condition much more serious, and that is malignant disease of the cervix uteri, which every now and then gives rise to symptoms much like those I have already described. As long as pregnancy does not exist the fact of bleeding a little more at the periods does not attract a woman's attention, but with the advent of pregnancy and the possible cessation of all bleeding for one or two months, and then its recurrence, makes a woman think she is going to have an abortion, and she consults her medical attendant, and if he does his duty he makes a vaginal examination and recognises the evil. If malignant disease of the uterus is found, I advise complete removal of the uterus, without emptying it, as a two or three months pregnant uterus can be removed per vaginam. I consider it is unwise to procure an abortion and then remove the uterus, and it is certainly not right to let the woman go on to full term with malignant disease of the uterus in order to remove a living child by abdominal section, and afterwards to remove a disease which has become more advanced. Of course, when I advise the complete removal of the uterus, it is under the distinct idea that the case is suitable for it and the only complication is pregnancy. Any woman who is pregnant and complains of bleeding from the vagina ought to be examined at once, so that proper treatment may be adopted. I have seen primary cancer of the vaginal wall cause great bleeding in an early case of pregnancy. Of course in some cases the woman does not think pregnancy exists, as she still has a more or less regular flow of blood *per vaginam*, the source being the ulcerating malignant surface and not true catamenial flow. A vaginal examination clears up the diagnosis, and treatment follows as a matter of course.

I have seen some cases of bleeding in pregnant women where the source was urethral, and also some cases of kidney bleeding have started at this time, and, of course, as an accidental complication, a bladder growth may exist which causes bleeding and complicates the diagnosis of pregnancy. It is not an uncommon thing for women to bleed from hemorrhoids during their pregnancy and not at other times, and sometimes in their ignorance they have stated the source to be vaginal. I have known of cases where the bleeding from the rectum has been supposed to threaten an abortion, when a proper examination revealed the true source of the hemorrhage. It is our duty in the vaginal hemorrhages of pregnant women to investigate its source by an examination of the rectum—urethra—bladder—kidney—cervix uteri or vaginal walls, and having put all these sources on one side, and seeing blood come from inside the os uteri, then to investigate its origin. For this, three chief points must be borne in mind:—(1) The persistence of one or more menstrual periods; (2) hemorrhage from *intra*-uterine gestation and possibly leading to abortion; (3) Hemorrhage as a symptom of *extra*-uterine gestation.

The first point I have already dealt with at the commencement of this paper. The other two points are of the greatest possible importance; viz., the differential diagnosis between intra- and extra-uterine pregnancy when the chief symptom is only that of uterine bleeding.

I have had under my care, or seen in consultation, some 25 cases of extra-uterine gestation, and some 500 cases of early abortion of intra-uterine pregnancy, and I can say even now that I regard the above problem as one of the most difficult I am asked to solve, and I wish to put before you the points I regard as being important. There is no organ in the female body that has caused so many mistakes as the uterus, and I have always before my mind the possibility of coming to grief over an early pregnant uterus. As an obstetrician and gynæcologist I am more likely to diagnose intra-uterine pregnancy when it does not exist than to fall into the opposite error; but the reverse mistake is more often made by my surgical friends, who have not the same horror of the uterus that I have. During the first three months the diagnosis of intra-uterine pregnancy rests almost entirely on the altered shape and position and softening of the uterus. All the other signs and symptoms are liable to be absent. I regard this uterine element in the diagnosis as of the highest importance, and I strongly advise all men who practice gynæcology to do what I did my first ten years at the Hospital; viz., to examine if possible every case of early pregnancy, so as to learn by the finger what no book can teach. You learn to recognise the peculiar softness of the os and cervix uteri, the altered shape of the uterine body; you feel resistance at points in the vaginal roof that you do not feel in the non-pregnant uterus; you notice the early increase of the body of the uterus in front of the cervix, as you push your finger high up; and then later you notice the increased bulging at the sides of the uterine body, and by degrees, if you examine the same woman at intervals of a week or ten days, you notice that sometimes one side of the uterus is more enlarged than the other, and that this alters again at your next examination. This is a very important point, as it often leads to the idea that there is a one-sided tumour of the uterus, or even an extra-uterine gestation sac. Hegar's sign of early pregnancy when present is very valuable, and depends on a marked softening of the lower uterine segment, by which it appears on a combined examination that the body and cervix are disconnected, the body frequently being mistaken for an independent tumour by those not aware of this sign. The point we have to determine in most cases is this: Is it a case of intra-uterine pregnancy with bleeding from the uterus, and probably threatening abortion ; or is it a case of extra-uterine pregnancy with uterine bleeding as a symptom? To solve this question you want a thorough knowledge of the symptoms of early pregnancy, both intra-uterine and extra-uterine, and also a knowledge of the physical and local conditions appertaining to both. It has often happened that an extra-uterine pregnancy has been overlooked, and all the symptoms put down to an ordinary abortion impending. It is of far less consequence to diagnose an extra-uterine pregnancy which does not exist than to miss one that does, provided always that the supposed extra-uterine pregnancy is not operated on.

The three chief points in the differential diagnoses of these conditions relate to the history, the pain and the bleeding. In my own experience the history does not help us much up to the time of pain and bleeding, and then the history how the pain and bleeding began is of importance. I need not refer to the ordinary symptoms of an extra-uterine gestation, nor compare it with many abdominal conditions that are liable to be mistaken for it, but I will simply consider the two symptoms, pain and bleeding, in their relation to extra- and intra-uterine pregnancy.

In my experience the **bleeding from the uterus** is more profuse in the *intra*-uterine pregnancy, and a profuse and continuous uterine bleeding would, I think, almost always negative extra-uterine pregnancy pure and simple. Yet one must bear in mind that many women abort with an ordinary intra-uterine pregnancy with very little loss of blood ; and these are the cases that may be mistaken for extra-uterine ones, where, as a rule, the bleeding is only slight. Again, in the extra-uterine cases, we see now and then a deciduous membrane passed, and this may be mistaken for an ordinary intra-uterine abortion. *Pain*, as a rule, is more severe in extra-uterine cases, and accompanied with more or less shock. Yet I have seen very severe pain and great shock in ordinary intra-uterine abortions, especially when there has been concealed intra-uterine bleeding before the external bleeding has become apparent.

I will briefly mention two cases of intra-uterine pregnancy and abortion, with symptoms of extra-uterine gestation :—

**Case 1.**—A. B., 2½ months pregnant, was taken with abdominal and uterine pains, and vaginal bleeding coming from inside the uterus. The vaginal examination showed the uterus to be equal in size to the time stated ; there was a big swelling in Douglas' pouch, and there was a history of the woman feeling sudden abdominal pain on lifting a heavy tub; the os uteri was slightly patent, and I fancied I felt an ovum high up. The case was diagnosed as intra-uterine pregnancy, with an impending abortion, and an intra-uterine blood tumour from accidental rupture of some pelvic vessel. In a few hours this diagnosis was proved to be correct, and the woman made a complete recovery unaided by any operation.

**Case 2.**—B. C., pregnant two months, taken with abdominal pain and distension, and vaginal bleeding. Uterus equal in size to supposed time of pregnancy, and a swelling to be felt to the left of the uterus in the position of the broad ligament. The diagnosis was that of intra-uterine pregnancy, with abortion impending, and a hemorrhage in the left broad ligament. The case was very difficult to diagnose, but I was greatly aided by two points, the size of the uterus, and the excessive uterine bleeding, for which I cleared out the uterine cavity of a two months' gestation, with a complete recovery of the patient.

These two cases, each with a suspicious history of extra-uterine pregnancy, were diagnosed chiefly by the size and position of the uterus corresponding to the supposed time of pregnancy. Of course, we all know that the uterus enlarges in cases of extra-uterine gestation, but all the cases I have seen have not given me the same idea that a normally pregnant uterus of the same date would. I am only now talking about cases not over three months. I remember one case of extra-uterine gestation where, at full time, labour pains set in, and we found the uterus equal to a seven months' pregnancy, and empty, the child being in the abdomen.

Here is one case where the symptoms pointed to intra-uterine pregnancy, with ordinary abortion; yet it was extrauterine, as shown by the operation, which was perfectly successful.

M. N. missed one period, began to bleed at what would be the second period, and at irregular intervals for a month, when not very severe but continuous pains set in, just like ordinary uterine pains; clots and bits of tissue-like membranes were passed. The os appeared more patent than usual, and under ordinary circumstances, from the history, one would have been justified in diagnosing an ordinary miscarriage; but the uterus was not equal to three months, and this made me a bit suspicious that it might be extra-uterine. And yet, as the bleeding had been going on for one month, I thought possibly the ovum ceased to develop then, and this would account for the size of the uterus being equal to two months and not three. At this time there was no definite swelling to be felt in the pelvis; but a few days after this visit I saw her again, after an attack of severe abdominal pain, when there was a post-uterine fulness distinctly to be felt. I diagnosed extra-uterine pregnancy, and operated successfully. The pregnancy was left Fallopian, and there was free blood in the pelvis.

These cases are very troublesome and cause us great anxiety. I am sure that no one can see many of them without wishing for a few more aids to their diagnosis.

---

